# Ectopic pregnancy: when is expectant management safe?

**DOI:** 10.1007/s10397-012-0736-6

**Published:** 2012-03-06

**Authors:** Sharon P. Rodrigues, Kirsten J. de Burlet, Ellen Hiemstra, Andries R. H. Twijnstra, Erik W. van Zwet, Trudy C. M. Trimbos-Kemper, Frank W. Jansen

**Affiliations:** 1 Department of Gynecology, K6-76, Leiden University Medical Center, PO Box 9600, 2300 RC Leiden, the Netherlands; 2 Department of Medical Statistics, Leiden University Medical Center, Leiden, The Netherlands

**Keywords:** Suspected ectopic pregnancy, Expectant management, Management, Beta-hCG, Cutoff

## Abstract

This study was conducted to evaluate expectant management in asymptomatic patients with an initial serum beta-hCG titer of <2,500 IU/l and to determine the independent ability of initial serum beta-hCG titers and trend of serum beta-hCG to predict successful expectant management. A cohort of patients (*N* = 418) with suspected ectopic pregnancy (EP) between January 1991 and July 2008 is described. Three groups were defined: group I (*n* = 182), immediate surgical intervention (<24 h); group IIa (*n* = 130), unsuccessful expectant management (surgical intervention during follow-up), and group IIb (*n* = 99), successful expectant management (spontaneous regression of trophoblast). Hospital protocol was not complied in 35 cases (Table 1). Beta-hCG levels >3,000 IU/l occur in our expectant management group; however, none of these cases were successful. Unnecessary surgery was prevented in 14% (*n* = 7) of asymptomatic patients with initial beta-hCG of >2,000 IU/l. The success rate of expectant management was 49%, without a rise in complication rate or number of acute cases. In conclusion, the initial serum beta-hCG cutoff level of 2,000 IU/l is not a rigid upper limit for accepting expectant management in suspected EP and best practice is case specific. In asymptomatic patients, the serum beta-hCG cutoff level of at least 2,500 IU/l can be used for expectant management. This cutoff could be higher, but interpretation is limited due to censure in follow-up inherent to the predefined clinical protocol. There is no gain in including patients for expectant management with initial serum beta-hCG level >3,000 IU/l.

## Introduction

Despite the availability of accurate diagnostic algorithms for the detection of ectopic pregnancy (EP), choosing the best treatment when EP is suspected can still pose a dilemma in daily practice. This dilemma arises in particular when patients without any clinical symptoms are diagnosed with EP. The latter tends to happen more often due to the availability of sensitive serum beta human chorionic gonadotrophin (beta-hCG) tests and high-resolution endovaginal ultrasonography that allows us to detect EP in early pregnancy even before clinical symptoms have the chance to set in [1, 2]. Consequently, early intervention has become a possibility and could potentially prevent serious complications. However, a considerable number of the EPs can also resolve spontaneously and treatment is not always necessary [3–5]. Inherently, early detection and quick intervention could result in overtreatment.

A recent cost analysis showed that diagnosing EP in a single visit could potentially save around £ 1 million per year in Scotland [6]. Because of this, the authors suggest that diagnosis of EP should be improved and that a single serum biomarker or other imaging modalities should be developed. However, this could also lead to a higher rate of overtreatment. Besides, the unnecessary exposure to risks in overtreatment and the expenses of this treatment could undo the gained savings. Therefore, modifications should ideally focus on predicting the need for future treatment earlier in the diagnostic pathway.

Various treatments for EP have been studied, i.e., surgical intervention (laparoscopy/laparotomy), medical treatment, and expectant management. Expectant management has only been advised in a selective group of patients, i.e., asymptomatic patients with relatively low (<2,000 IU/l) and diminishing serum beta-hCG levels [4, 5, 7, 8]. However, for over 10 years, asymptomatic patients with suspected EP and an initial serum beta-hCG titer below 2,500 IU/l were treated with expectant management. This study was designed to evaluate this policy and to determine the independent ability of initial serum beta-hCG titers and trend of serum beta-hCG titers in the prediction of successful expectant management.

## Methods

**Fig. 1:**
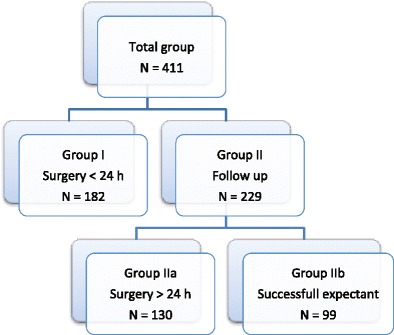
Categorization of patients in groups according to their management type. *Group I* immediate treatment (within 24 h) by surgical intervention; *Group IIa* unsuccessful expectant management (surgery >24 h); *Group IIb* successful expectant management

This study describes a cohort of patients with suspected EP between January 1991 and July 2008 at the Leiden University Medical Center, Leiden, The Netherlands. Suspected EP was defined as patients with a serum beta-hCG above 1,500 IU/l and no ultrasonographic signs of intrauterine pregnancy or patients with abdominal pain and/or blood loss combined with a positive urine pregnancy test (i.e., beta-hCG >50 IU) without signs of intrauterine pregnancy. Clinical symptoms, ultrasonography results, and serum beta-hCG values of these patients were registered. Patients with a heterotopic pregnancy or medical treatment of EP were excluded. After inclusion, patients were categorized according to their management type: either in the group of immediate treatment (within 24 h) by surgical intervention (group I) or in the group of expectant management when follow-up of beta-hCG and clinical signs took place (group II; Fig. 1). Some patients in group II required surgery during follow-up (group IIa; unsuccessful expectant management), while others had a spontaneous regression of throphobast (group IIb; successful expectant management).

Data analysis was performed with SPSS 16.0. Patient characteristics were compared with Chi-square/Cramer’s V, except for age, which was analyzed with one-way ANOVA. Beta-hCG values were compared for every group combination with the Mann–Whitney test. For the comparison of complications between the intervention groups, patients in group I who presented with pain were excluded because of a presumed association between acute presentation (symptoms of acute pain and/or peritoneal irritability objectified by the treating physician) and higher complication rates. Furthermore, their acute presentation was not preventable, in contrast to patients in group IIa. For patients in group IIa, the choice to initially refrain from surgery includes a risk of future acute presentation and thus potentially an increased risk of complications. Therefore, patients with pain in group IIa were not excluded for comparison of complications (Chi-square/Cramer’s V).

To compare the course of serum beta-hCG levels among the groups, the ratio of each consecutive beta-hCG concentration to the initial concentration was calculated. The ratios in group IIb (successful expectant management) were compared to the ratios in the group IIa (unsuccessful expectant management) at predetermined time points. Therefore, the ratio at the time of surgery (group IIa) was compared to the highest ratio during follow-up (group IIb) (independent samples *t* test).

The optimal beta-hCG cutoff was determined for asymptomatic patients using a receiver operating characteristics (ROC) curve. Patients with hemodynamic instability or presenting with acute pain at initial presentation were excluded from this analysis because surgical intervention was obliged irrespective of the serum beta-hCG values. Success rate of expectant management was determined as the percentage of successes (IIb) of the total expectant management group (II). Finally, a logistic regression model was fitted for the probability of receiving surgery at some time during the diagnostic pathway based on initial serum beta-hCG level and an increase or decrease in beta-hCG between the initial measurement and the second measurement.

## Results

In the present study, 416 patients with suspected EP were identified. Five patients with a heterotopic pregnancy were excluded. None of the patients had medical treatment. Altogether, 411 patients were included (Fig. 1), of whom 182 patients needed surgical intervention within 24 h (group I) because of hemodynamic instability and/or acute pain (*N* = 149) or a serum beta-hCG level exceeding 2,500 IU/l (*N* = 33). Initially, 229 patients were asymptomaic and therefore eligible for expectant management (group II). Surgical intervention during follow-up was required in 130 patients because of no signs of spontaneous resolution, excessive pain, or hemodynamic instability (group IIa). In 99 patients, the EP regressed spontaneously (group IIb). During laparoscopy, no EP could be identified in one case (group I) which during follow-up culminated in an intrauterine gravidity. In all other surgical cases, EP was confirmed with pathology findings. In group IIb, a spontaneous abortion could not be excluded in 16 cases; however, EP remained the final diagnosis.

The distribution of the initial serum beta-hCG levels of asymptomatic patients in the three management groups shows that hospital protocol was not complied in 35 cases (Table 1). One patient with an initial serum beta-hCG level of 1,809 IU/l had a laparoscopy within 24 h because ultrasonography showed a vital EP. And 34 patients with initial serum beta-hCG levels above 2,500 IU/l were managed expectantly of whom 2 (5.5%) were managed expectantly with success (serum beta-hCG levels, 2,735 and 2,799 IU/L). Table 2 displays general characteristics of the three groups. Patient characteristics were similar, whereas initial serum beta-hCG titers differed significantly. Both intervention groups (from group I only patients without symptoms) had a comparable number of laparotomies and conversion rates. Neither time nor length of surgery differed significantly. Furthermore, the incidence of acute presentation and complication rates were similar in both groups, except for conversion due to heavy blood loss which occurred once in group I and not in group IIa (Table 3).

**Table 1:** Distribution of initial serum beta-hCG levels

Initial beta-hCG titer	Group I	Group IIa	Group IIb	Total
	Immediate intervention	Unsuccessful expectant management	Successful expectant management	
<2,000	1	88	92	181
2,000–2,500	0	10	5	15
>2,500	32	32	2	66
Total	33	130	99	262

Initial serum beta-hCG distribution of patients without severe pain or hemodynamic instability in the three management groups

**Table 2:** Group characteristics

	Group I	Group IIa	Group IIb	*P*
	Immediate intervention	Unsuccessful expectant management	Successful expectant management	
N	182	130	99	
Age (years) ^a^	31.9 ± 4.8	31.4 ± 4.4	31.7 ± 5.5	NS
Parity ^b^	0 (0–5)	0 (0–4)	1 (0–4)	NS
Previous abortions ^b^	0 (0–7)	0 (0–2)	0 (0–3)	NS
Previous miscarriages ^b^	0 (0–6)	0 (0–10)	0 (0–10)	NS
History tubal pathology ^c^	33 (18%)	34 (26%)	19 (19%)	NS
History infertility ^c^	44 (24%)	42 (32%)	19 (19%)	NS
Amenorrhea at first visit (weeks) ^a^	6.30 ± 1.6	6.26 ± 1.9	6.18 ± 2.1	NS
Number of days till second visit ^a^	N/A	2.6 ± 1.6	2.8 ± 2.2	NS
Initial beta-hCG level (IU/l) ^d^	3834 (89–100414)	1403 (58–20858)	530 (34–2799 IU/l)	<.001
Observational period (days) ^d^	N/A	6.3 (2–21)	28.8 (3–95)	

General characteristics of the three management groups

*NS* not significant, *N/A* not applicable

^a^Mean ± SD

^b^Median (range)

^c^Number of cases (percentage of intervention group)

^d^Mean (range)

**Table 3:** Characteristics and complications of the intervention groups

	Group I (*N* = 33)	Group IIa (*N* = 130)	*P*	Test
	Immediate intervention	Unsuccessful expectant management		
Laparotomies^a^	4	11	NS	Cramer’s V
Time surgery took place	Time of surgery during the day		NS	Cramer’s V
Length of surgery (hh:mm)^b^	01:02 ± 00:03	01:04 ± 00:02	NS	Mann–Whitney
Tubal rupture ^a^	0	1	NS	Cramer’s V
Persistent trophoblast ^a^	0	9	NS	Cramer’s V
(No decline of beta-hCG levels)				
Conversions ^a^	2	9	NS	Cramer’s V
Heavy blood loss (>1 l) ^a^	1	2	NS	Cramer’s V
Conversions due to heavy blood loss ^a^	1	0	<.05	Cramer’s V

Comparison of the characteristics and complications of the two intervention groups. As explained in the “Methods” section, only asymptomatic patients of group I are analyzed (because of a presumed association between acute presentation and higher complication rates), whereas in group IIa, all patients are analyzed (because an increased risk is a direct consequence of expectant management)

^a^ *N*

^b^Mean ± SD

The ROC curve did not indicate an optimal trade-off between sensitivity and specificity for successful expectant management. The best trade-off reached was at a serum beta-hCG cutoff level of 1,275.5 IU/L with a sensitivity of 65.3% and specificity of 87.1%. None of the successful expectant management cases had an initial beta-hCG level above 3,000 IU/l, while there were cases with an initial beta-hCG level above 3,000 in the unsuccessful group (IIa).

The calculated mean ratio, based on the highest beta-hCG value during follow-up in group IIb, was 1.11 (99% confidence interval (CI), 0.99–1.22). The mean ratio, based on the beta-hCG value which indicated surgery in group IIa, was 2.53 (99% CI, 1.56–3.50), which is significantly higher (*p* = <.001).

Success rate of expectant management was 49%. In 14% (*n* = 7) of the cases with an initial serum beta-hCG above 2,000 IU/l, expectant management was successful (Table 1). However, there were also nine cases in this group who presented with severe symptoms during follow-up and therefore needed surgery. Of these nine cases, seven had an initial serum beta-hCG level above 2,500 IU/l (six above 3,750 IU/l and one of 2,671 IU/l). The other two had initial beta-hCG levels of 2,360 and 2,364 IU/l, respectively. If an initial beta-hCG cutoff level of 2,000 IU/l had been used for expectant management, success rate would have been 50%.

**Fig. 2:**
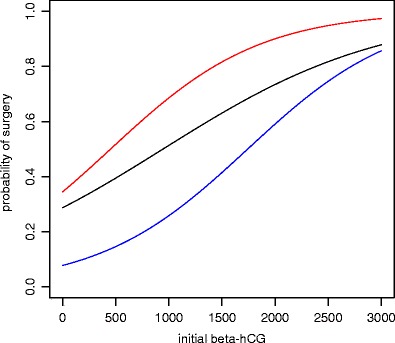
Estimated probability of surgery. The *black line* represents the first visit. The *red line* indicates patients with a rising beta-hCG at the second visit, and the *blue line* indicates patients with a declining beta-hCG

Figure 2 illustrates the estimated probabilities of eventual surgery at a given beta-hCG level as a result of the logistic regression model. The second visit is taken into account by defining a dummy variable which indicates a rising (red line) or declining (blue line) beta-hCG. The figure shows for example that if an 80% chance of surgery is used as cutoff, theoretically, patients with an initial serum beta-hCG level of 2,400 IU/l or higher should have surgery and patients with an initial serum beta-hCG level below 2,400 IU/l are eligible for follow-up. Using a cutoff of 90% certainty at the second visit, according to this model, surgery is advised in patients with an initial serum beta-hCG level of 2,000 IU/l or higher which is rising, while a declining beta-hCG level under these restrictions could be interpreted as acceptable.

## Discussion

An initial serum beta-hCG cutoff level of 2,500 IU/l for expectant management can be used for asymptomatic patients with suspected ectopic pregnancy. This is also suggested by a small prospective study by Lurie et al. [9] in which patients with rising serum beta-hCG levels were successfully managed expectantly and a serum beta-hCG cutoff level of 2,500 IU/l was used. However, the current protocol of the Dutch Society of Obstetrics and Gynecology states that expectant management is justified in asymptomatic patients with a serum beta-hCG value until 2,000 IU/l. By raising beta-hCG cutoff from 2,000 to 2,500 IU/l in our clinic, unnecessary surgery was prevented in 14% of the patients eligible for expectant management, while complication rates and the number of patients with acute presentation are comparable in both intervention groups.

Previous studies have used receiver operating characteristics (ROC) curves to determine an optimal serum beta-hCG cutoff level for diagnosis of EP (2,000 IU/l in asymptomatic patients) [1]. These studies do not consider spontaneous resolution of EP. We also aimed to determine an optimal serum beta-hCG cutoff level for expectant management in asymptomatic patients suspected of EP with a ROC curve. The result shows that a serum beta-hCG cutoff level of 1,275.5 IU/L gives the optimal trade-off between sensitivity and specificity. However, the results of this (and other similar) ROC curve(s) should be interpreted with caution because of the design of the study. When serum beta-hCG levels rise above 2,500 IU/l, patients become eligible for laparoscopy and follow-up of serum beta-hCG concentration is censured. Although the probability that these cases would become acute is presumed to be very high, the true outcome remains unknown. To determine the true optimal cutoff, ideally patients should be managed expectantly until symptoms of acute pain and/or peritoneal irritability arise. However, exposing patients to such potential risks would be unethical, and thus, such a study is unlikely to ever be done.

The success rate of expectant management in this study was 49%. Previous studies have shown great variety in success rates (46.7–92%) [4, 5, 8, 10]. This variety is most likely explained by differences in inclusion criteria and protocols used. Selection criteria for expectant management in previous studies were much stricter. Most studies only include patients with low and stable or decreasing serum beta-hCG levels. In our study, patients with increasing serum beta-hCG concentration were allowed to remain expectant and the cutoff level of initial serum beta-hCG was 2,500 IU/l, which is higher than generally used in previous studies. Logically, a higher cutoff level of beta-hCG will lead to a lower sensitivity explaining a higher number of patients with an unsuccessful expectant policy. However, this protocol also leads to a higher specificity resulting in lower numbers of unnecessary surgery. Altogether in this study, seven patients (14%) were spared unnecessary surgery without observing an overall increase in complications or acute presentation. In our opinion, this justifies the lower success rate.

Korhonen et al. have shown a rise in serum beta-hCG in 49% of the patients requiring surgical intervention [8]. This supports our observation in serum beta-hCG trend, i.e., increase in beta-hCG ratio in the unsuccessful expectant group and decrease in the successful expectant management group. In the unsuccessful expectant management group, the mean beta-hCG ratio at intervention was 2.5 (99% CI, 1.56–3.50), whereas at the highest point, the mean beta-hCG ratio was 1.1 (99% CI, 0.99–1.22) in the successful expectant management group. This finding suggests that if expectant management is chosen for, a rise of initial beta-hCG concentration by one and a half should be interpreted as an indication for surgical intervention. Of course, the before mentioned censure in follow-up when serum beta-hCG levels rise above 2,500 IU/l also influences this result. However, we reasoned that prolonged follow-up of these patients would probably lead to an even higher serum beta-hCG level. By respecting lower levels, we are likely to stay on the safe side of the limit, but we should keep in mind that these limits are not rigid. Table 2 illustrates that the upper limit of our protocol was not obliged rigidly. Serum beta-hCG levels even above 3,000 IU/l occur in our expectant management group. However, none of these cases were successful. Furthermore, 67% (*N* = 6) of the cases with an initial serum beta-hCG level above 2,000 IU/l, who also developed acute symptoms during follow-up, had an initial serum beta-hCG above 3,000 IU/l. This implies that there is absolutely no gain in including patients for expectant management when initial serum beta-hCG levels are above 3,000 IU/l.

Since EP is expensive to diagnose and exclude, mainly due to the many assessment visits needed [6], determining the appropriate treatment early in the diagnostic pathway could potentially save a lot of costs. The logistic regression model could be indicative in early decision making. However, because the model is drawn up solely from the presented cohort, conclusions about absolute cutoff levels cannot be made. Interpretation of the calculated probabilities should be left in the hands of the gynecologist with knowledge of the specific case. Overall, the model shows that an increase in serum beta-hCG level at the second visit strongly raises the probability of having surgery during follow-up. Although a decrease of serum beta-hCG level at the second measurement evidently lowers the chance of culminating into an acute surgical case, these patients should still be observed with caution, especially in those with a high initial serum beta-hCG level (i.e., >2,500 IU/l) who, despite a decreasing serum beta-hCG level during follow-up, still have a high chance of culminating into an acute situation. This is also illustrated by the published cases of patients with rupture of EP while serum beta-hCG level is disappearing [11, 12]. A recent study showed that a serum beta-hCG level of >1,500 IU/l is associated with a higher rate of tubal rupture than a beta-hCG level of <1,500 IU/l [13]. Therefore, frequent follow-up and clear patient instructions are necessary for safe expectant management.

Although today medical treatment with methotrexate is gaining popularity, as it can be a good alternative for surgery, this was not in the scope of the current study. Multiple-dose systemic treatment with methotrexate has been shown to be as effective as surgical treatment, whereas single-dose treatment is less effective. However, health-related quality of life was more severely impaired after systemic methotrexate. Furthermore, systemic multiple-dose treatment is only cost-effective in patients with a serum beta-hCG level of <3,000 IU/l, or a single-dose treatment in patients with a serum beta-hCG level of <1,500 IU/l [7, 14, 15]. Because of these reasons, in some clinics, such as the study clinic, methotrexate as treatment of EP has not been implemented as standard treatment.

## Conclusion

The often advised initial serum beta-hCG cutoff level of 2,000 IU/l should not be handled as a rigid upper limit for accepting expectant management in asymptomatic patients with suspected ectopic pregnancy. However, there seems to be no gain in including patients for expectant management with initial serum beta-hCG levels above 3,000 IU/l. Because of the heterogeneity in patient profiles, the best treatment option is case specific. Besides the clinical symptoms and initial serum beta-hCG level, the second serum beta-hCG measurement (i.e., rising or declining) and trend in serum beta-hCG concentration are also very indicative for the management choice.
